# *In Vitro* Infection of Human Nervous Cells by Two Strains of *Toxoplasma gondii*: A Kinetic Analysis of Immune Mediators and Parasite Multiplication

**DOI:** 10.1371/journal.pone.0098491

**Published:** 2014-06-02

**Authors:** Nour Mammari, Philippe Vignoles, Mohamad Adnan Halabi, Marie Laure Darde, Bertrand Courtioux

**Affiliations:** 1 National Institute of Health and Medical Research 1094, Tropical Neuroepidemiology Institute, Limoges, France; University of Limoges, National Center for Scientific Research France 3503 Institute of Genomic, Environment, Immunity, Health and Therapy, Limoges, France; 2 National Center for Scientific Research France 7276, France 3503 Institute of Genomic, Environment, Immunity, Health and Therapy, University of Limoges, Faculty of Pharmacy, Limoges, France; 3 Universitary Hospital, Department of Parasitology, Biological Resource Centre for Toxoplasma, Limoges, France; Centre National de la Recherche Scientifique, France

## Abstract

The severity of toxoplasmic infection depends mainly on the immune status of the host, but also on the *Toxoplasma gondii* strains, which differ by their virulence profile. The relationship between the human host and *T. gondii* has not yet been elucidated because few studies have been conducted on human models. The immune mechanisms involved in the persistence of *T. gondii* in the brains of immunocompetent subjects and during the reactivation of latent infections are still unclear. In this study, we analyzed the kinetics of immune mediators in human nervous cells *in vitro*, infected with two strains of *T. gondii*. Human neuroblast cell line (SH SY5Y), microglial (CMH5) and endothelial cells (Hbmec) were infected separately by RH (type I) or PRU (type II) strains for 8 h, 14 h, 24 h and 48 h (ratio 1 cell: 2 tachyzoites). Pro-inflammatory protein expression was different between the two strains and among different human nervous cells. The cytokines IL-6, IL-8 and the chemokines MCP-1 and GROα, and SERPIN E1 were significantly increased in CMH5 and SH SY5Y at 24 h pi. At this point of infection, the parasite burden declined in microglial cells and neurons, but remained high in endothelial cells. This differential effect on the early parasite multiplication may be correlated with a higher production of immune mediators by neurons and microglial cells compared to endothelial cells. Regarding strain differences, PRU strain, but not RH strain, stimulates all cells to produce pro-inflammatory growth factors, G-CSF and GM-CSF. These proteins could increase the inflammatory effect of this type II strain. These results suggest that the different protein expression profiles depend on the parasitic strain and on the human nervous cell type, and that this could be at the origin of diverse brain lesions caused by *T. gondii*.

## Introduction

The majority of human *Toxoplasma* infections in immunocompetent hosts are asymptomatic, After an acute infection, tachyzoites can escape from the immune system, leading to the formation of tissue cysts containing bradyzoites, especially in the brain. However, in the immunocompromised host, latent bradyzoites in cysts revert to tachyzoites, leading to reactivation of chronic toxoplasmosis and development of a toxoplasmic encephalitis [1]. Thus, the severity of *Toxoplasma* infection obviously depends on the host immune status.

The role of the *Toxoplasma* strain is more debated. Genotyping of *Toxoplasma* isolates from all continents revealed a complex population structure. Up to now, 15 haplogroups were described [2]. These haplogroups comprise the 3 main clonal lineages initially described (type I, II and III) and other haplogroups that cluster various atypical strains and new clonal lineages [3], [4] [5] [6]. On the basis of lethality in mice, type I strains were classified as virulent, and type II and III as non-virulent. These 3 types differ with respect to their ability to transmigrate across cellular barriers during invasion. Type I strains exhibit a higher migratory capacity than type II strains [7]. In humans, the influence of the strain in the clinical outcome is obvious in the severe cases of toxoplasmosis in immunocompetent patients due to the most divergent strains such as those circulating in the Amazonian forest [6], Its role is also highly suspected in the higher occurrence and severity of ocular toxoplasmosis in South America [8]. But it remains unclear if the *Toxoplasma* strain has any influence on the development of brain infection. In a study performed on 88 immunocompromised patients, the distribution of type II vs non-type II strains was not significantly different when patients were stratified by underlying cause of immunosuppression, site of infection (cerebral or extra-cerebral), or outcome [9].

During a toxoplasmic infection, the immune response can firstly reduce the parasite proliferation during acute infection, and then maintains chronic infection in immunocompetent hosts. During acute infection, monocytes, neutrophils, and dendritic cells are recruited to the site of infection [10] [11] [12]. These cells also play a role for migration and dissemination of the parasite in peripheral tissues and the central nervous system (CNS). This process depends on the parasitic strain. The type II strains induce superior migration of infected dendritic cells compared to type I strains [7]. Experimental data on animal models suggest that the immune response type 1 (Th1) is activated against *T. gondii* to control parasite replication. This immune response leads to production of interferon-gamma (IFN-γ) in mice infected with RH (type I) or ME49 (type II) strains [10] [13]. IFN-γ is the major mediator of resistance to *T. gondii* in the murine model; it can inhibit parasite replication, preventing toxoplasmic encephalitis during the late stage of infection in mice. During this host response, other cytokines and chemokines are produced, which can promote infiltration of immune cells to the site of infection [14] [15] [16].

The early events that enable the parasite to cross the blood-brain barrier are poorly understood. Different experimental studies have demonstrated that brain endothelial cells infected with RH (type I), ME49 (type II) and PRU (type II) strains of *T. gondii* express ICAM-1, IL-6, and MCP-1[17] [18] [19] [20]. These studies have reported a possible role of these cytokines in parasite invasion into the brain. After infection with the RH strain, mouse endothelial cells upregulated E-selectin, P-selectin and ICAM-1 expression, known to support the migration of immune cells to sites of inflammation [21] [22] [23]. After invasion step, *T. gondii* can infect microglial cells [24] [14], astrocytes [25] [14] [26] and neurons [16] [26] leading to cyst formation [14].

In the mouse brain, microglial cells play a major role in the control of infections caused by *T. gondii* type II. These cells inhibit efficiently parasite growth and may thus function as important inhibitors of *T. gondii* propagation within the CNS by IFN-γ and NO-independent mechanisms [24] [27]. *In vitro* comparison has shown that astrocytes are infected more efficiently than neurons and microglia [28] [27]. This is confirmed by *in vivo* experiments demonstrated that astrocyte is the predominant cell type infected by RH and PRU strains in the brain [26] [29]. Murine astrocytes have also been shown to inhibit *in vitro* the growth of NTE (type II) *Toxoplasma* strain [25]. Astrocytes infected by ME49 strain become activated to produce IL-1, IL-6 and GM-CSF [14]. These pro-inflammatory proteins together with microglia-produced cytokines play an important role in inducing infiltration of immune cells into the brain. In addition, Lüder et *al*. found that rat astrocytes and neurons are suitable host cells for the intra-cerebral proliferation of PLK strain (type II) [27]. It was described that both tachyzoites and bradyzoites can deregulate the function of murine neurons [30]. IFNγ and IL-6 production by murine neurons indicates that these cells contribute to intracellular control of type II and I strains [31] [32] [33].

Most of these results were obtained in the murine model. The role of human neuro-endothelial cells in neuro-invasion by the parasite and the behavior of human microglial cells and neurons after *T. gondii* infection remains to be shown.

In this study, we analyzed the production of immune mediators during different times following *Toxoplasma* infection in a human model. For that, three human brain cells: human microglial, human endothelial and a human neuroblast cell lines were used. These cells were infected *in vitro* by different *T. gondii* strains. Proteomic analysis and parasite quantification were performed at each time point of infection to identify the various pro-inflammatory proteins produced by human nervous cells infected with different *T. gondii* strains and their impact on parasitic growth.

## Materials and Methods

### Ethics statement

This study was carried out in strict accordance with the recommendations in the Guide for the Care and Use of Laboratory Animals of the National Institutes of Health. The protocol was approved by the Committee on the Ethics of Animal Experiments of Limousin, France (Permit Number: 3-07-2012). All efforts were made to minimize suffering.

### Parasite strains

Two strains of *Toxoplasma gondii* parasite were used: RH strain, a type I strain highly virulent in mice (DL90<10), and PRU strain, a type II strain with a non-virulent profile in mice (DL100 = 10^3^) [34].

### Cyst isolation

PRU cysts were obtained from the brains of Swiss mice infected one month earlier. Infected mouse brains were extracted and homogenized by several passages through a 20-gauge needle in Modified Eagle's Medium (MEM) 0.1% (Gibco, Cergy Pontoise, France) Tween 80 (Sigma-Aldrich, Lyon, France), to disrupt tissues. PRU cysts were isolated by Percoll gradient [35]. Briefly, ninety milliliters of Percoll (Amersham Biosciences, Orsay, France) were rendered isotonic by adding 10 mL of 10× MEM. The pH was neutralized by adding HCL 10%. Brain homogenates were diluted in MEM, 0.1% Tween-80%; 1.5 mL of 30% Percoll and then 1.5 mL of 90% Percoll were successively added with a tapered Pasteur pipette under suspension. After centrifugation (1811 g), cysts were recovered in the 30% Percoll layer. The cyst suspension was centrifuged to eliminate the remaining Percoll, and the pellet was recuperated in MEM, 0.1% Tween-80. PRU bradyzoites were released from cysts by trypsin 1X (Gibco) at 37C° for 10 min and recovered after centrifugation (1811 g–15 min).

### Tachyzoite production

RH tachyzoites from mouse ascites and PRU bradyzoites released from cysts were inoculated in human fibroblastic cell cultures (MRC5). After parasite multiplication, tachyzoites from both strains were released by disruption of infected cells by passages through 20 and 27 gauge needles. Cell debris were removed by filtration through polycarbonate membrane 3 µM (Nucleopore Whatman, Versailles, France). Purified parasites were suspended in MEM and enumerated.

### Human nervous cell lines

- Microglial cells (CMH5): Human microglial cells (CMH5) cells (kindly provided by Pr P. Vincendeau, Bordeaux, France) [36] [37] were grown in Dulbecco's Modified Eagle Medium (DMEM, Gibco), supplemented with 10% fetal bovine serum (FBS) inactivated at 56°C for 1 h, 0.2 mM sterilized L-cysteine (Sigma-Aldrich), 2 mM L-glutamine (Gibco), 2 mM sodium pyruvate (Gibco), 100 UI/m streptomycin-penicillin (Gibco), 2 mM Hepes (Gibco) and 0.1 mM β- mercaptoethanol (Sigma-Aldrich). CMH5 cells were seeded in culture flasks (25 cm^2^) at a density of 5×10^6^ cells/mL. Confluent cells were trypsinized in DMEM.

-Endothelial cells (Hbmec): Human bone marrow endothelial cells (Hbmec) were obtained from the cell line established by D. Paulin (University of Paris 7, France), K. Schweitzer (Free University of Amsterdam, The Netherlands), and B. Weksler (Weill Medical College of Cornell University, UK) [38], [39] and [40]. The properties of these cells are similar to those of endothelial brain cells, with respect to endothelial adhesion molecules, cell surface markers, and morphologic characteristics. Hbmec cells were grown in DMEM, supplemented with 10% FBS inactivated at 56°C for 1 h, 2 mM L-glutamine, 2 mM sodium pyruvate, 100 UI/mL of streptomycin-penicillin, 10 mM Hepes buffer, 4.5 g/mL glucose, 3500 UI/mL heparin and 0.15 mg/mL gentamicin. Hbmec cells were seeded in culture flasks (25 cm^2^) pre-treated with 2% gelatin at density of 4×10^6^ cells/mL. Confluent cells were trypsinized in PBS.

- Neuroblastoma cells (SH SY5Y): This human neuroblast cell line from neural tissue (SH SY5Y) (kindly provided from Pr M.O. Jauberteau-Marchan, Limoges, France) [41] is used in most of the researches on Alzheimer's disease [42] and presents all the characteristics of neurons. SH SY5Y were grown in RPMI 1640 medium (Gibco), supplemented with 10% FBS inactivated at 56°C for 1 h, 2 mM L-glutamine, 2 mM sodium pyruvate, 100 UI/mL streptomycin-penicillin. SH SY5Y cells were seeded in culture flasks (25 cm^2^) at density of 4×10^6^ cells/mL. Confluent cells were detached by Versen 1X (Gibco).

All cell lines were cultured at 37°C in humidified air containing 5% CO_2_.

### Parasite-cell co-cultures

The required number of cells were cultured 48 h before infection, time needed for cell adhesion. After cell adhesion, 6×10^6^ cells/mL of CMH5 were infected by 1.2×10^7^ tachyzoites/mL. For Hbmec and SH SY5Y, 4×10^6^ cells/mL were infected by 8×10^6^ tachyzoites/mL (ratio: 1 cell/2 tachyzoites) of RH or PRU strains for 8 h, 14 h, 24 h and 48 h. Uninfected control cells were cultured under the same conditions. All co-cultures were performed separately in culture flasks (25 cm^2^). The experiments were performed four times, in the same conditions but at different times to ensure reproducibility of results. Infections with different strains were performed at different periods to avoid any contamination between strains.

After infection, supernatants were recovered and frozen at −80°C; cells were trypsinized for 10 min at 37°C. After centrifugation at 804 g for 10 min, cell pellets were recovered and frozen at −80°C.

### Cytokine, chemokine & growth factor analysis

Cytokine, chemokine & growth factor production profiles were detected by Proteome Profiler array kit, type human cytokines array panel A (R&D, Lille, France). Each nitrocellulose membrane contains duplicated spots of 36 different antibodies anti-cytokines, chemokines, growth factors and adhesion proteins. The kit was used according to manufacturer's instructions. Each membrane was incubated with a mixture chemiluminesence substrate A&B (Promega, Lyon, France) and exposed to G-BOX (Syngene, Versailles, France). The image was captured using GeneSnap software (Syngene). Results appear as dark spots, protein expression levels were determined by quantifying the intensity of coloration, expressed as pixel units, using GeneTools software (Syngene). The negative control quantification value was subtracted from the quantification value of each spot.

### RNA extraction & quantification of *T. gondii* burden by semi-quantitative reverse transcription polymerase chain reaction (RT-PCR)

RNAs from each co-culture pellet were extracted using the RNeasy Mini Kit (Qiagen, Essones, France), according to manufacturer's instructions. DNAc was used to quantify the viable parasite burden in each co-culture by amplifying a *T. gondii* 529 pb repeat gene [43] [44]. qRT-PCR was performed using one step SYBR Green RT-PCR Kit (Qiagen). Each RNA sample (≤100 ng) was added to PCR tubes containing SYBR master mix (12.5 µL), RT Mix (0.25 µL), specific primers (Sigma-Aldrich) 529 pb (0.6 µM) sense: 5′AGGCGAGGTGAGGATGA3′; anti-sense: TCGTCTCGTCTGGATCGAAT 3′ [44] and sterile water for final volume of 25 µL. Following assay optimization, negative samples were used. qRT-PCR was performed with a Rotor-Gene 6000 (Qiagen) using cycling conditions: reverse transcription 10 min at 55°C, PCR initial activation 5 min at 95°C, denaturation 5 sec at 95°C and annealing/extension 10 sec at 60°C for 40 cycles. Quantification was performed using a range of tachyzoites from 5×10^4^ to 1 *Toxoplasma* amplified by primers for the 529 pb gene. In addition, to verify the stage obtained after PRU culture in MRC5 cells, BAG-1, a bradyzoite-specific gene, was amplified by qRT-PCR using BAG-1 primers sense: 5′-TGA GCG AGT GTC CGG TTA TT-3′ and anti-sense: 5′-ATT CCG TCG GGC TTG TAA T-3′ [45] (Sigma-Aldrich).

### Statistical analysis

All experiments were performed four times and values of corrected pixels were obtained from pixel values of infected cells minus pixel values of uninfected cells. They were expressed by ± mean standard error. Statistical analysis was performed using a parametric ANOVA test and Tukey HSD (Honestly Significant Difference) test for multiple comparisons of means, with 95% confidence level. A value of *p*<0.05 was considered statistically significant.

In each kinetic study, the comparison of immune mediator production was performed between three groups: cells, strain and infection times. This was carried out firstly between each variable of each group: cell (CMH5 *vs* SH SY5Y) (SH SY5Y *vs* HBMEC) (HBMEC *vs* CMH5), strain (RH vs PRU) and idem for each period of infection. All interactions between the three groups were also studied (two by two).

The parasite burdens were evaluated as the ratio of final tachyzoite number/initial tachyzoite number. When the ratio was less than 100%, the tachyzoite mortality was higher than their proliferation. Based on the fact that 1 RH strain tachyzoite gives 2 tachyzoites after 7 h [46], 128 tachyzoites must be obtained after 48 h which represents a maximal ratio of 12800% parasite burden. Data on parasite multiplication rate were analyzed by Tukey HSD test, with 95% confidence level. A value of *p*<0.05 was considered statistically significant.

## Results

For all infected cells by PRU strain, semi-quantitative RT-PCR for BAG-1 gene shown absence of bradyzoites in the inoculum.

### 1. Kinetics of pro-inflammatory proteins and *T. gondii* burden in infected human endothelial cells (Hbmec)

**Figure 1:**
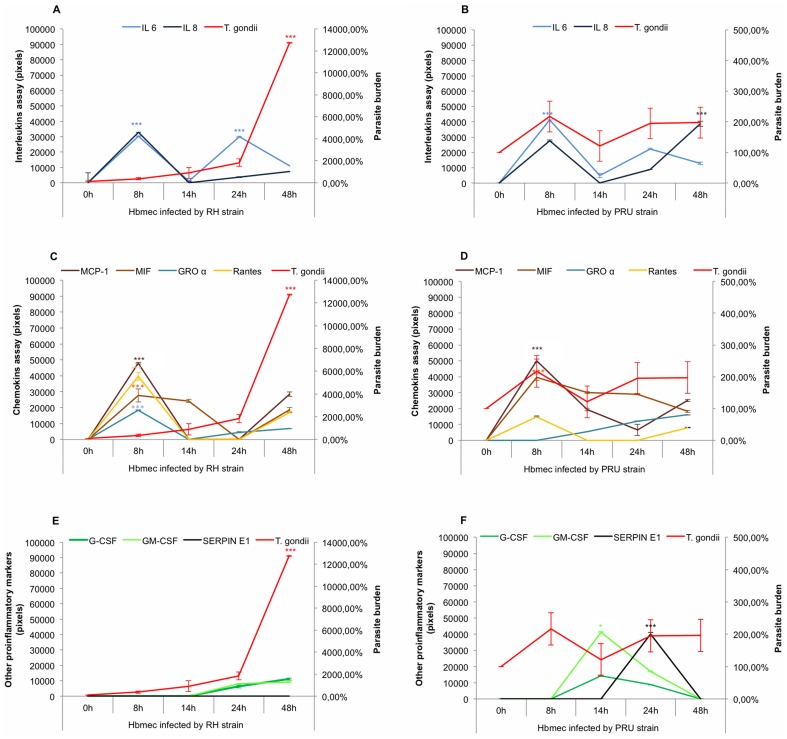
Kinetics of pro-inflammatory proteins synthesized by infected Hbmec. A and B: cytokine expression profiles; C and D: chemokine expression profiles; E and F: growth factor and SERPIN E1 expression profiles were determined after 8 h, 14 h, 24 h and 48 h infection by two strains of *T. gondii* RH (Figure 1A, 1C and 1E) and PRU (Figure 1B, 1D and 1F). A basal expression level for each pro-inflammatory protein *T. gondii* burden was determined at T_0_. Concentrations of immune mediators were calculated using values of corrected pixels obtained from uninfected cells (negative control) subtracted from pixel values of infected cells. *T. gondii* burdens were evaluated by the percentage of *Toxoplasma* multiplication, knowing that the parasite burden at T_0_ is 100%. The parasite burden scale was multiplied 100 times (Only in Hbmec infected by RH strain). *p<0.05, ** *p*<0.01 and *** *p*<0.001.

**Figure 2:**
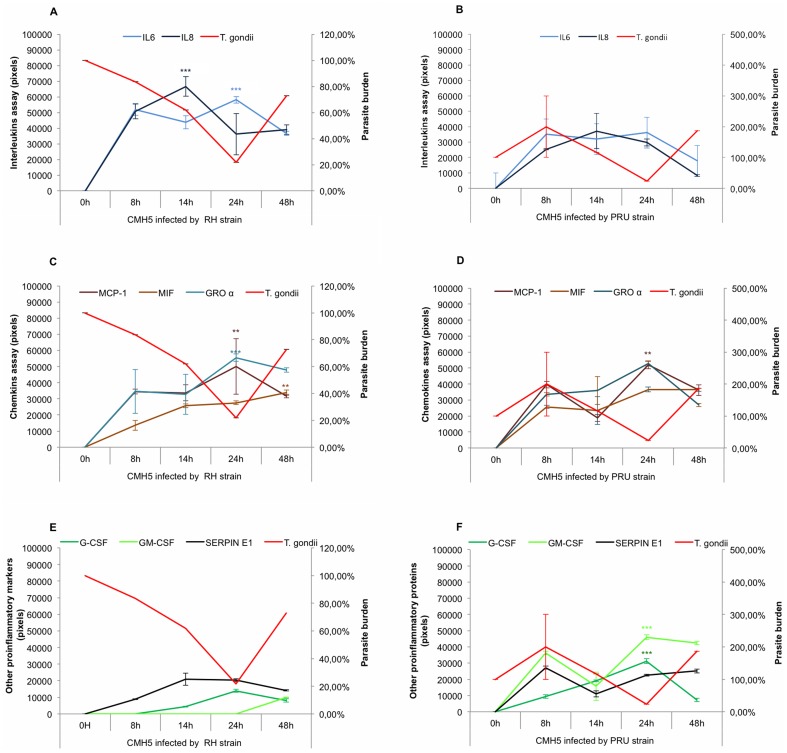
Kinetics of pro-inflammatory proteins synthesized by infected CMH5. A and B: cytokine expression profiles; C and D: chemokine expression profiles; E and F: growth factor and SERPIN E1 expression profiles were determined after 8 h, 14 h, 24 h and 48 h infection by two strains of *T. gondii* RH (Figure 2A, 2C and 2E) and PRU (Figure 2B, 2D and 2F). A basal expression level for each pro-inflammatory protein *T. gondii* burden was determined at T_0_. Concentrations of immune mediators were calculated using values of corrected pixels obtained from uninfected cells (negative control) subtracted from pixel values of infected cells. *T. gondii* burdens were evaluated by the percentage of *Toxoplasma* multiplication, knowing that the parasite burden at T_0_ is 100%. *p<0.05, ** *p*<0.01 and *** *p*<0.001.

As shown in Figure 1A, 1C, 1E, Hbmec infected with RH strain showed a basal expression level for interleukins, chemokines and other inflammatory proteins in endothelial cells at T_0_. Expression of IL-6 and IL-8 in endothelial cells reached peak levels at 8 h post infection. Production of interleukins declined at 14 h post infection. At 24 h post infection, IL-6 increased significantly whereas IL-8 remained at a low level compared to 8 h (Figure 1A). Chemokines: MCP-1, MIF, GROα and Rantes reached peak levels significantly at 8 h post infection (Figure 1C). Expression of all chemokines dropped between 14 h and 24 h post infection and then increased non significantly (NS) at 48 h post infection (Figure 1C). G-CSF and GM-CSF production increased slightly from 14 h to 48 h post infection (NS) (Figure 1E). In the period when all protein levels declined, the RH parasite burden rose sharply in endothelial cells (Figure 1A, 2C).

At 8 h post infection with PRU (Figure 1B, 1D, 1F), IL-6, IL-8, MIF, MCP-1 synthesis and parasite levels peaked (Figure 1B, 1D), when GROα and Rantes synthesis remained low (Figure 1D). From 8 h to 14 h post infection, all immune mediators decreased significantly except GROα and the parasite burden increased slightly (NS) (Figure 1B, 1D). Between 14 h and 48 h post infection, IL-6, IL-8, MCP-1, GROα (NS), Rantes (NS) synthesis and the parasite burden (NS) increased (Figure 1B, 1D). On the other hand, G-CSF (NS) and GM-CSF expression profiles reached peaks at 14 h (Figure 1F). SERPIN E1 increased significantly between 14 h and 24 h of infection whereas the parasite burden was constant (Figure 1F).

### 2. Kinetics of pro-inflammatory proteins and *T. gondii* burden in infected human microglial cells (CMH5)

CMH5 infected with RH showed a basal expression level for interleukins, chemokines and other inflammatory proteins in microglial cells at T_0_ (Figure 2A, 2C, 2E). The expression levels of interleukins (IL-6 and IL-8), chemokines (MCP-1, MIF and GROα) and growth factors (G-CSF and GM-CSF) increased steadily from 8 h until 24 h post infection whereas the parasite burden decreased erratically until 24 h post infection (Figure 2A, 2C, 2E). At 48 h post infection, all protein levels decreased except SERPIN E1 synthesis, which increased (NS) (Figure 2E). The parasite burden reached a peak at 48 h post infection, but this increase was not statistically significant.

After infection of CMH5 with PRU, at 8 h post infection, we observed a non-significant synthesis for interleukins (IL-6 and IL-8), chemokines (MCP-1, MIF and GROα), growth factors (G-CSF and GM-CSF) and SERPIN E1. The parasite burden reached peaks at 8 h post infection (Figure 2B, 2D, 2F). At 14 h post infection, MCP-1 and growth factor levels slumped (Figure 2D, 2F) and the parasite burden decreased slightly (NS) until 24 h post infection. At this time post infection, all protein levels reached their peaks. At 48 h post infection, all protein levels decreased except SERPIN E1 (NS) and the parasite burden levels increased (NS) (Figure 2F).

### 3. Kinetics of pro-inflammatory proteins and *T. gondii* burden in an infected human neuroblastoma cell line (SH SY5Y)

**Figure 3:**
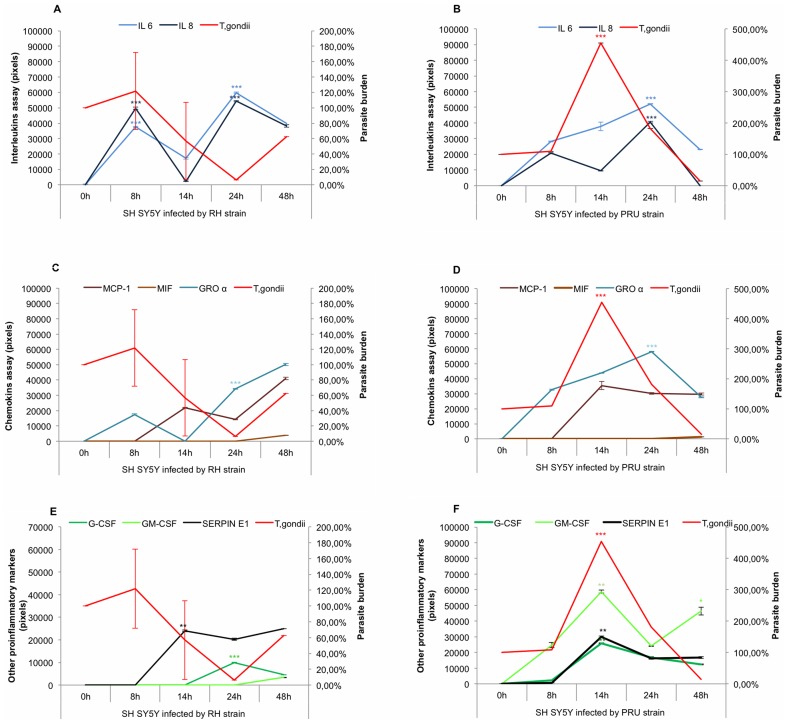
Kinetics of pro-inflammatory proteins synthesized by infected SH SY5Y. A and B: cytokine expression profiles; C and D: chemokine expression profiles; E and F: growth factor and SERPIN E1 expression profiles were determined after 8 h, 14 h, 24 h and 48 h infection by two strains of *T. gondii* RH (Figure 3A, 3C and 3E) and PRU (Figure 3B, 3D and 3F). A basal expression level for each pro-inflammatory protein *T. gondii* burden was determined at T_0_. Concentrations of immune mediators were calculated using values of corrected pixels that were obtained from pixel values of uninfected cells (negative control) subtracted from pixel values of infected cells. *T. gondii* burdens were evaluated by the percentage of *Toxoplasma* multiplication, knowing that the parasite burden at T_0_ is 100%. *p<0.05, ** *p*<0.01 and *** *p*<0.001.

As shown in Figure 3A, 3C, 3E, SH SY5Y infected with RH showed a basal expression level for interleukins, chemokines and other inflammatory proteins in neurons at T_0._ Expression of IL-6 and IL-8 in neurons reached peak levels significantly and the parasite burden fell (NS) from 8 h post infection. The level of IL-6 and IL-8 dropped at 14 h post infection, when the parasite burden was also significantly lower compared to the parasite burden at 8 h post infection. At 24 h post infection, interleukin synthesis increased significantly and the parasite burden dropped sharply. At 48 h post infection, interleukin synthesis declined while the parasite burden rose gradually (NS) (Figure 3A). Among the chemokines (MCP-1, MIF and GROα) (Figure 3C), only GROα synthesis was significant at 24 h post infection and the parasite burden decreased between 8 h and 24 h post infection (NS) (Figure 3C). At 48 h post infection, all chemokines and parasite burden increased (NS) (Figure 3C). In addition, a low level of G-CSF was produced significantly at 24 h post infection (Figure 3E). SERPIN E1 was expressed from 8 h to 48 h (Figure 3E).

For PRU infection (Figure 3B, 3D, 3F), IL-6 and IL-8 synthesis increased significantly between 8 h to 24 h post infection, whereas the parasite burden reached a peak significantly at 14 h and then decreased sharply until 48 h. After 24 h post infection, both synthesis of interleukins and parasite burden fell compared to 24 h post infection (Figure 3B). Expression of MCP -1 and GROα, as well of G-CSF, GM-CSF and SERPIN E1 showed similar profiles (Figure 3D, 3F). Growth factors and SERPIN E1 synthesized proteins decreased significantly after 24 h post infection compared to 14 h post infection, when the parasite burden reached its lowest level.

### 4. Comparison of pro-inflammatory protein expression profiles in human microglial cells (CMH5), human endothelial cells (Hbmec) and human neuroblastoma cell line (SH SY5Y) infected by RH and PRU strains

To compare these results, a statistical analysis was performed. MCP-1 synthesis at 24 h post infection was significantly higher in CMH5 (Figure 2C, 2D) compared to Hbmec (*p*<0.004) (Figure 1C, 1D) and SH SY5Y (*p*<0.0001) (Figure 3C, 3D). GROα and IL-6 were less produced by Hbmec (Figure 1A, 1B, 1C, 1D) compared to CMH5 (*p*<0.0001) (Figure 2A, 2B, 2C, 2D) and SH SY5Y (*p*<0.0001) (Figure 3A, 3B, 3C, 3D). Synthesis at 24 h post infection was significantly higher compared to other infection times (*p*<0.0001). Furthermore, MIF was significantly produced only by CMH5 and Hbmec cells. Which was present in higher amount in CMH5 at 24 h post infection (*p*<0.0001) (Figure 2C, 2D) and very low in SH SY5Y (*p*<0.001) (Figure 3C, 3D). Hbmec infected with *Toxoplasma* produced Rantes (*p*<0.0001) (Figure 1C, 1D) and sICAM-1 (*p*<0.0001) (data not shown).

Rantes synthesis was high in Hbmec infected by RH at 8 h post infection (Figure 1C). IL-8 expression was high in CMH5 infected by RH (*p*<0.0001) at 24 h post infection compared to 14 h (*p*<0.0001) and 48 h (*p*<0.0001) (Figure 2A). In the presence of PRU, all cells expressed high level of G-CSF (*p*<0.002) and GM-CSF (*p*<0.0001) (Figure 1F, 2F, 3F). G-CSF and GM-CSF expression was significantly increased at 24 h post infection in CMH5 (*p*<0.0001) (Figure 2F). Compared to the RH strain, the PRU strain stimulates Hbmec to produce SERPIN E1 (*p*<0.001) (Figure 1F).

## Discussion

Different experimental approaches were used to analyze expression of immune mediators during *T. gondii* infection in brain cells. For example, regarding human brain cells, Xiao et *al*, used a human neuroepithelioma cell line (neural cells, line SK-N-MC) infected with 3 canonical *T. gondii* strains to show differences in a wide range of biological functions such as the immune response [47]. They noticed a modulation of the expression of pro-inflammatory gene like IL-8 depending of the strain type.

The originality of our work resides in the comparison of the different protein levels of cytokines, chemokines and other immune factors in three human nervous cells infected *in vitro* by RH (type I) and PRU (type II) *Toxoplasma* strains during the 48 first hours post-infection by tachyzoites. Proteome Profiler Arrays allowed analyzing simultaneously a broad spectrum of pro-inflammatory proteins.

In immunocompetent mice and *in vitro* models, tachyzoites can infect astrocytes [25] [14] [26], microglial cells [24] [14] and neurons [16] [26] leading to cyst formation [14]. In addition, astrocytes, microglial cells and neurons are suitable for the persistence of cysts in chronically infected rats [27].

In this study, we identified many pro-inflammatory factors like cytokines, chemokines, and growth factors secreted by different human nervous cells after *Toxoplasma* infection. All these factors have roles in the host immune response to parasite challenge: chemokines and their receptors are important in the control of parasite replication and CNS inflammation, and trigger pro-inflammatory responses to many microbial pathogens [15]. Among the pro-inflammatory proteins, were chemokines (MCP1, GROα, MIF and Rantes) designed to recruit immune cells and cytokines (IL-6 and IL-8) able to activate immune responses. Growth factors (G-CSF and GM-CSF) are not only hematopoietic colony-stimulating factor but also neuronal growth factors with strong anti-apoptotic actions on neurons. In parallel to this pro-inflammatory protein synthesis, we observed a repeatable decrease in parasitic burden for each *T. gondii* strain at 24 h post infection in microglial cells and neurons. This suggests that this decrease may be due to production of various immune mediators involved in the early control of *T. gondii* multiplication in human nervous cells. At 48 h post infection, parasite burden increased in a large part of infected cells, in RH-infected cells whatever the cell type. We also observed a lysis of cells, which could explain the decrease of immune response.

Endothelial cells constitute the blood-brain barrier through which the tachyzoites pass into the CNS. Our results show that these cells are stimulated with *Toxoplasma* to produce immune mediators mainly at 8 h post infection. This reaction rapidly decreases between 14 h and 48 h post infection, when parasite burden increased. This suggests that these cells do not react strongly against *Toxoplasma* infection compared to neurons and microglial cells. The production of interleukins and chemokines in the early hours of the infection might activate the immune response. In these cells, PRU strain induces more inflammatory response than RH strain, as shown by a higher secretion of chemokines and growth factors between 14 h and 48 h. Endothelial cells specifically express sICAM-1 in the presence of PRU strain, but not after infection with RH strain (data not shown). Barragan et *al*. demonstrated that this soluble form of ICAM, sICAM-1, has an inhibitory effect on transmigration of *T. gondii* across BeWo (human placenta), Caco2 (human intestine) and MDCK (canine kidney) cells, whereas it did not significantly affect host cell invasion by the parasite [19]. According to these results, in our model, sICAM-1 could inhibit the transmigration process of type II strain through brain endothelial cells.

After infection by both *T. gondii* strains, all cells (microglial cells, endothelial cells, and neurons) synthesized mainly IL-6, IL-8, MCP-1 and GROα. In CMH5 and SH SY5Y, the expression of cytokines, chemokines and growth factors was variable but significantly higher at 24 h post infection, the time when parasite burden (RH and PRU) declined. At 24 h post infection, microglial cells infected by both strains produced high levels of MCP-1. This chemokine can participate in the control of *T. gondii* infection as described in human fibroblastic cells (MRC5) [48] and human astrocyte cells [49]. *In vivo*, it increases inflammation, promoting recruitment of monocytes and lymphocytes. The immune responses could be amplified in these cells by secretion of GROα. Synthesis of GROα was also significantly higher at 24 h post infection in microglial cells compared to other cells. This chemokine has a major role in neutrophil activation. Kikumura et *al*. [50] showed that GROα was up-regulated in the retina and brain of infected mice and involved in neutrophil infiltration. Thus, microglial cells seems to be major cerebral immune cells, able to amplify immune reactions against the parasite, promoting production of the main chemokines.

IL-6 and IL-8 production was predominant in microglial cells and neurons compared to endothelial cells, especially at 24 h post infection, which might suggest that these two interleukins have a role in controlling proliferation of RH and PRU strains in microglial cells and neurons. IL-6 is known to control parasite burden and inflammatory activity, in the brain together with other cytokines (such as IL-10) [14]. IL-6 is also a mediator responsible for the production of acute phase proteins and increases cytotoxic activity of NK cells. For the first time, we demonstrated a local production for IL-8 in human nervous cells that appeared to have a crucial role in neutrophil recruitment to the brain as described in patients with retinochoroiditis. IL-8 has a role in the inflammatory mechanisms of acute toxoplasmic retinochoroiditis [51]. It is noteworthy that mice lacking CXCR2, the high affinity receptor of IL-8, show a significant increase in tachyzoite proliferation and a large number of cysts in the brain [52].

Besides the previously described chemokines and interleukins, infected microglial cells produced high levels of MIF compared to other cells; this production can boost the control of parasite proliferation in these cells. MIF has an essential role in controlling *T. gondii* type II proliferation and the severity of inflammation in a mouse model [53].

G-CSF and GM-CSF were produced by all cells, especially those infected by PRU strain. In addition to their role in growth, differentiation and maturation of macrophages, monocytes and dendritic cells, they have the capacity to inhibit *in vivo* neutrophil apoptosis, and the ability to lyse tachyzoites *in vitro* in the presence of specific antibodies [54]. G-CSF and GM-CSF may thus extend the inflammatory process in the brain by enhancing phagocytosis [55] and cytotoxic activities [56]. Infected astrocytes and endothelial cells also become activated to produce GM-CSF [57]. Studies using *T. gondii* infection in mice, which can cause CNS inflammation, reported a detrimental role for GM-CSF by increasing parasitic burden in peritoneal macrophages [58]. Therefore, GM-CSF is a potent pro-inflammatory cytokine that plays a pathogenic role in the CNS inflammatory disease, experimental autoimmune encephalomyelitis [59], suggesting that G-CSF and GM-CSF may amplify the pro-inflammatory immune reaction during PRU infection in brain.

For the first time, we showed the up-regulation of serine protease inhibitor (SERPIN E1) in infected human nervous cells. It occurred particularly in neurons and microglial cells a few hours post infection. Many members of the serine protease inhibitor superfamily play an important role in physiological and pathological processes, and can be regarded as protease inhibitors, which are involved in coagulation reactions, fiber dissolution, angiogenesis, complement activation, and immune and inflammatory reactions. It has been suggested that SERPINs (SERPIN B3, B4) could inhibit host cell apoptosis that may inhibit replication and decrease *T. gondii* viability. Therefore, the persistence of up-regulated SERPIN E1 in microglial cells and neurons could also be involved in limiting the growth of *T. gondii* and preventing death of infected human nervous cells. In addition, SERPIN E1, plasminogen activator inhibitor 1, inhibits the uPA/uPAR pathway which promotes active MMP-9 forms secreted by *Toxoplasma* infected macrophages [60], which could limit the invasion of immune cells infected by *T. gondii* into the brain.

In addition to several studies which have shown that murine and rat microglial cells contribute *in vitro* to the inhibition of *T. gondii* replication [27], our results suggest that both human microglial cells and neurons can react against *Toxoplasma* by producing IL-6, IL-8, MCP-1, GROα and SERPIN E1 [20] [17] [18]. These immune mediators may play a role in the control of *Toxoplasma* multiplication at 24 h post infection in the human brain, as shown by the decrease in parasitic burden at this time of cell infection. G-CSF and GM-CSF were specifically produced in all cells infected with PRU, suggesting a pro-inflammatory effect of this strain in human nervous cells compared to RH strain. In this work we did not detect a production of IFN-γ in infected human nervous cells. This could be explained by the lack of interaction between the effector immune cells. For this reason, this work will be complemented by a study of IFN-γ effect on immune mediators and on parasite multiplication rate in each infected cells.
